# Cross-sectional imaging of intestinal barrier dysfunction by confocal laser endomicroscopy can identify patients with food allergy in vivo with high sensitivity

**DOI:** 10.1038/s41598-021-92262-4

**Published:** 2021-06-17

**Authors:** Timo Rath, Walburga Dieterich, Christiane Kätscher-Murad, Markus F. Neurath, Yurdagül Zopf

**Affiliations:** 1 Department of Gastroenterology, Ludwig Demling Endoscopy Center of Excellence, University Hospital Erlangen, Friedrich-Alexander University Erlangen-Nuernberg, Ulmenweg 18, Erlangen, Germany; 2 Department of Gastroenterology, Hector-Center for Nutrition, Exercise and Sports, University Hospital Erlangen, Friedrich-Alexander University Erlangen-Nuernberg, Erlangen, Germany; 3 Deutsches Zentrum Für Immuntherapie DZI, Friedrich-Alexander University Erlangen-Nuernberg, Erlangen, Germany

**Keywords:** Diseases, Gastroenterology, Medical research

## Abstract

Food allergy (FA) affects approximately 3 to 4% of the adult population in westernized countries. Suspected FA is even more prevalent and requires extensive diagnostic work-up. Within this study, we evaluated whether assessment of the integrity of the epithelial barrier by confocal laser endomicroscopy (CLE) during colonoscopy can be used as a screening tool to identify patients with FA. 60 patients with suspected FA were prospectively included. Serology with total and food-specific IgE, anti-tissue transglutaminase, skin prick testing, food intolerance tests, food intake registration and assessment of clinical complaints were performed. During colonocopy, standardized CLE was performed in the terminal ileum and at two colorectal sites. Analysis of CLE images included functional (i.e. presence of epithelial barrier dysfunction) and quantitative parameters of intestinal architecture. 27 of 60 patients (45%) were diagnosed with FA. Barrier dysfunction was analyzed on 65.837 ileal and on 93.251 colonic images. 96% of patients with FA exhibited functional and structural barrier defects while barrier dysfunction was found in only 33% of patients without FA (*p* < 0.0001). Visualizing barrier dysfunction with CLE for in vivo diagnosis of FA had a sensitivity and specificity of 96% and 67%, respectively, with a positive and negative prediction of 70% and 96%, respectively. Parameters intrinsic to the crypt architecture including crypt diameter, intercrypt distance, crypt lumen diameter and colonic vasculature were not different between patients with and without FA. CLE-based imaging of the intestinal barrier during colonoscopy might help in stratifying patients with suspected FA for further diagnostic work-up.

## Introduction

Self-reported adverse reactions to food reaches up to one fifth of the population^1^, and although prevalence of food allergy (FA) as diagnosed by objective measures is lower, FA remains a common disease. As such, a cross-sectional study on 333.200 pediatric patients in the US estimated a prevalence of FA of 6.7% in children^2^, while in adults FA affects approximately 3% to 4% of the population in westernized countries^3^. As a consequence, FA represents a considerable socioeconomic burden to the healthcare systems: as estimated in a recent study, overall costs of FA only in children reach up to $24.8 billion dollar per year in the US^4^, taking into account also adult patients, the economic burden of allergic reactions caused by food and anaphylaxis was an estimated half a billion dollars in 2007^5^. Furthermore, the global prevalence of food allergies seems to be increasing^6–8^, thereby potentially further boosting the costs associated with diagnosis and treatment of FA. At the same time, diagnosis of FA is challenging. Commonly used allergy tests such as skin prick testing or serum–radioallergosorbent tests (RASTs) exhibit only limited sensitivity and specificity^9^ and although measurements of total and allergen specific IgE levels are recommended to identify the causative food allergen, they are not diagnostic themselves^9^. Therefore, oral food challenge (OFC) with increasing doses of a potential antigen over a defined period of time remains the diagnostic the gold standard FA^9,10^. However, since OFC is resource-intense and requires vigilant cooperation between patient, physician, and nutritionist, it is only very infrequently used outside specialized centres^8^. Given the constraints, novel diagnostic tests that can be readily applied and sensitively identify patients with suspected FA hold the potential to facilitate the diagnostic work-up.

Local assessment of the intestinal barrier with advanced endoscopic technologies such as confocal laser endomicroscopy (CLE) as a technology enabling microscopic imaging in the GI tract in vivo might be used for rapid detection of mucosal alterations in FA. Here, we used CLE to visualize barrier dysfunction in patients with suspected food allergy under regular diet and specifically explored whether CLE can be used as a screening tool to identify patients with FA in real time.

## Material and methods

### Patients

Patients presenting with suspected food allergy in our outpatient department were prospectively included. Diagnostic work-up of suspected food allergy included serologic testing for total and food-specific IgE, skin prick testing, food intake registration, assessment of complaints according to the Rome IV criteria^11^ as well as gastroenterological investigation including serology for celiac disease, breath testing for lactose, fructose and sorbitol intolerance and bacterial overgrowth, stool sample analysis and esophagogastroduodenoscopy and colonoscopy. Diagnosis of FA was based on serologic testing, clinical symptoms and oral food challenge. The study was approved by the ethics committee of the Friederich-Alexander University Erlangen-Nuernberg and all patients gave their written informed consent prior to study inclusion.

### Colonoscopy and confocal laser endomicroscopy

All patients received bowel preparation with low-volume PEG-based bowel lavage in a split dose regimen. Colonoscopy was performed using commercially available HD endoscopes and video processors (EC38-i10 and Optivista EPK-i7010, both Pentax Medical, Tokyo, Japan). Care was taken to minimize suction and endoscope trauma. For confocal imaging, a dedicated CLE imaging system consisting of a portable laser station (Cellvizio) and confocal miniprobes (ColoFlex UHD, Mauna Kea Technologies, Paris, France) was used. CLE imaging was performed in a standardized fashion at three sites during colonoscopy. After reaching the terminal ileum, 5 mL Fluorescein 10% were intravenously injected as a contrast agent. Afterwards, the CLE probe was positioned under endoscopic guidance onto the mucosa of the terminal ileum, cecum and rectosigmoid junction and low-powered blue laser light of a wavelength of 488 nm was activated for tissue illumination. At each site, a CLE video of approximately 2 min was recorded with an image acquisition rate of 8 frames/per second. All pCLE images for each patient were stored on an external harddrive and were independently reviewed by two expert readers blinded to the clinical results of the patients. Analyses of the videos included functional parameters (i.e. integrity of the epithelial barrier in the terminal ileum and the colon) and morphometric assessment of the crypts and vasculature.

**Figure 1:**
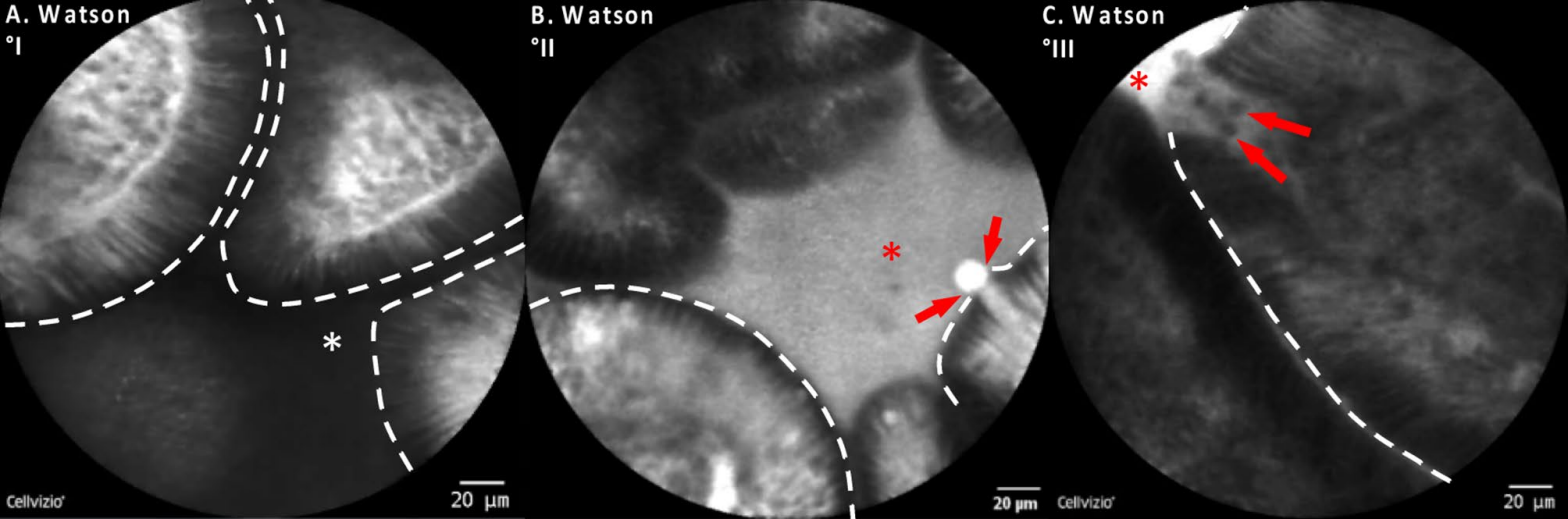
Semiquantitative grading of intestinal barrier dysfunction in the terminal ileum under CLE imaging. (**A**) Healthy terminal ileum (Watson Grade I): Healthy terminal ileum and intact epithelial barrier is characterized by finger-shaped villi with enterocytes forming the epithelial barrier. The intestinal lumen is dark when the intestinal barrier is intact (white asterisk). (**B**) Functional barrier (Watson Grade II) defect is characterized by shedding of single enterocytes generating a gap or loss of epithelial integrity with fluorescein leakage (red arrows) into the intestinal lumen which then turns bright (red asterisk). (**C**) Structural barrier defect (Watson Grade III) is characterized by shedding of multiple epithelial cells (red arrows) and fluorescein leakage into the lumen which as a consequence of fluorescein efflux turns bright (red asterisk). Dashed white line: apical border of the enterocytes forming the epithelial barrier.

Barrier dysfunction in the terminal ileum was assessed using the semi-quantitative Watson score into three grades^12–16^: (I) *intact epithelial barrier* with no fluorescein leakage; (II) *functional barrier defect* with shedding of single epithelial cells and fluorescein leakage into the intestinal lumen; (III) *structural barrier defect* with shedding of multiple epithelial cells, exposure of the lamina propria to the lumen and fluorescein leakage into the lumen (Fig. 1). Barrier dysfunction in the colon was assessed using a dichotomous distinction as previously described^13,17,18^: intact epithelial barrier in the colon was characterized by a crypt opening that appeared as a dark center in the crypt. During colonic barrier dysfunction, fluorescein leaked into the crypt lumen; therefore, the lumen was brighter than the surrounding epithelium.

### Cryptometric assessment of colonic architecture by CLE

For cryptometry, measurements were performed blinded to the clinical results of the patients at a time where no final diagnosis (food allergy versus no food allergy) was established yet. 10 frames per patient representative of the colonic architecture were analyzed. The following intrinsic architectural parameters of the crypts were calculated: (1) *intercrypt distance*, as the distance between the geometrical centers of neighboring crypts, as defined by the crypt lumen (2) *total crypt diameter*, defined as the distance between epithelial cells constituting the crypts measured in 2 axes crossing the centers of the crypts; (3) *crypt lumina diameter*. These parameters were measured using the IC-Viewer software version 3.8.6 (Mauna Kea Technologies, Paris, France). Regarding the vasculature in the colon, mean, minimal and maximal vessel diameter were measured using the vessel detection plug in of the IC-Viewer which enables automatic detection of the vessels directly from each endomicroscopic recording frame based on fluorescence intensity detection. Detection threshold was manually set at 10 μm for vessel detection.

### Endpoints, sample size and statistical analysis

The primary endpoint of this study was to assess the diagnostic performance of barrier dysfunction for the in-vivo diagnosis of food allergy. Sample size calculation was based on a recent study reporting barrier dysfunction with CLE in 70% of patients with IBS^19^. Assuming a prevalence of barrier dysfunction in 70% in patients with FA with a 2-sided significance of 0.05 and a power of 80%, a total of 57 subjects will be required at an enrolment ratio of 1 to 1 (FA vs. no FA). As the secondary endpoint, cryptometric parameters between patients with and without food allergy were compared. Statistical analysis was performed using Graph Pad Prism (GraphPad Software, San Diego, USA). Normal distribution of the data was tested using the Kolmogorov–Smirnov test and visualization of histograms. Failing to meet criteria for normal distribution, data were analysed using non-parametric tests. Epidemiological and clinicopathologic data are presented as means ± standard deviation (SD) and range. Dichotomous variables were compared using two-tailed Fisher’s exact test. A 2-sided *p* < 0.05 was considered significant.

### Ethics approval and informed consent

The study was approved by the ethics committee of the Friedrich-Alexander University Erlangen-Nurnberg and all patients gave their written informed consent prior to study inclusion. The study was conducted in accordance to the ethical guidelines of the Declaration of Helsinki.

## Results

### Clinical characteristics of the patient cohort

Between 2017 and 2019, a total of 60 patients presenting with suspected food allergy were prospectively included. In 27 of these patients (45%) with a mean age of 41 years (range 22–76), FA was diagnosed based on serology in combination with clinical symptoms and oral food challenge while in 33 patients (mean age 49 years, range 22–75), FA was ruled out. As shown in Table 1, patients with FA had significantly higher serum IgE levels (*p* = 0.00003) whereas serological expression of C-reactive protein (CRP), eosinophilic cationic protein (ECP), TNF, tryptase, histamine and diaminooxidase (DAO) as well as leukocyte counts were not different between patients with and without FA. Out of these 33 patients without FA, the following diagnoses were made after diagnostic work: microscopic colitis (n = 2), sorbitol intolerance (n = 2), histamine intolerance (n = 3), eosinophilic gastroenteritis (n = 1). Clinical and serologic characteristics of the patients are shown in Table 1.

**Table 1:** Clinical characteristics of the patient cohort.

	No food allergy (n = 33)	Food allergy (n = 27)	*p* value
**Baseline demography**			
Sex (m/%)	7/21%	6/22%	0.92
**Age**			
Mean ± SD	49.1 ± 13.9	41.3 ± 14.3	0.038
Range	22–76	22–75	
**Laboratory parameters**			
**Leukocyte count [× 10**^**9**^**/L]**			
Mean ± SD	6.8 ± 1.6	6.1 ± 1.8	0.12
**Serum CrP [mg/L]**			
Mean ± SD	1.3 ± 1.1	1.7 ± 1.4	0.40
**Serum IgE [kU/L]**			
Mean ± SD	39.0 ± 34.1	108.7 ± 59.5	0.00003
**Serum ECP [ng/mL]**			
Mean ± SD	11.4 ± 11.8	8.6 ± 5.4	0.93
**Serum tryptase [µg/mL]**			
Mean ± SD	5.1 ± 4.2	4.4 ± 2.5	0.55
**Serum TNF [pg/mL]**			
Mean ± SD	9.9 ± 6.4	8.3 ± 4.8	0.45
**Serum histamine [pg/mL]**			
Mean ± SD	0.43 ± 0.35	0.35 ± 0.38	0.56
**Serum DAO [U/mL]**			
Mean ± SD	13.8 ± 5.6	10.8 ± 7.7	0.21

### Endoscopy and barrier dysfunction in patients with and without Food allergy

All patients underwent gastroduodeno- and colonoscopy using high definition white light endoscopy (HD-WLE). During colonoscopy, pCLE was performed in a standardized procedure at three sites with approximately 2 min of video recording at the terminal ileum, cecum, rectosigmoid junction. At these sites, physical biopsies were obtained in parallel and processed for histopathological analysis in separate containers. In all patients, HD-WLE endoscopy was unremarkable.

From all patients, a total of 65.837 images from the terminal ileum were analyzed for the presence of barrier dysfunction, mean number of images per patient was 1097.

**Figure 2:**
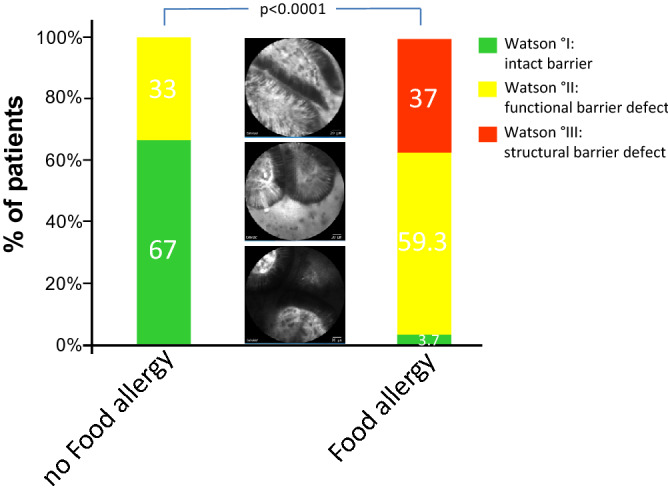
Barrier dysfunction in the terminal ileum in patients with and without Food Allergy. Overall, 26 out of 27 patients (= 96%) in the FA group exhibited barrier dysfunction in the terminal ileum under functional imaging with pCLE. In detail, 10 patients out of 27 patients (= 37%) exhibited a structural barrier dysfunction (Watson III) and 16 patients out of 27 patients (= 59.3%) had a functional barrier defect (Watson II). In the non-FA group, 11 out of 33 patients (= 33%) had barrier dysfunction, all of which were functional barrier defects. Differences in barrier dysfunction between patients with and without FA were statistically significant (*p* < 0.0001). Middle panel: Representative images of the different grades of barrier dysfunction on pCLE; from bottom to top: Watson I, Watson II, Watson III.

**Figure 3:**
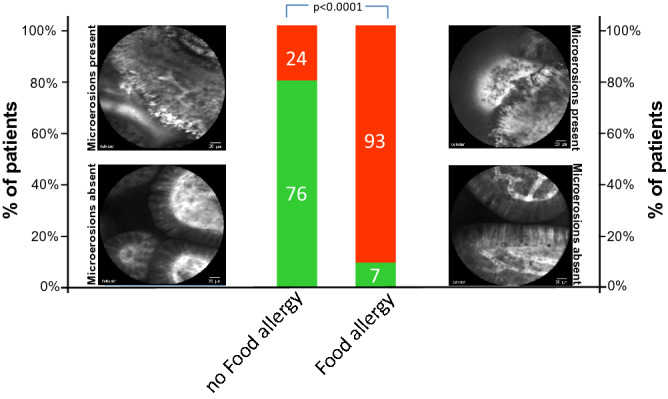
Microerosions in the terminal ileum in patients with and without Food Allergy. Microerosions, defined as an exposure of the lamina propria to the lumen with multiple cells being shed per site, were found in 25 out of 27 patients (92.6%) with FA. In the non-FA group, 8 out of 33 patients exhibited microerosions in the terminal ileum (24.2%). Differences in the prevalence of microerosions between patients with and without FA were statistically significant (*p* < 0.0001). Red segment of each bar: percentage of patients with microerosions, green segment of each bar: percentage of patients without microerosions.

Using this approach, fluorescein leakage was significantly more common in patients with food allergy compared to patients without food allergy: As shown in Fig. 2, 26 out of 27 (= 96%) patients with confirmed FA exhibited barrier dysfunction in the terminal ileum under functional imaging with pCLE while only 11 out of 33 patients (= 33%) in the non-FA group had barrier dysfunction (*p* < 0.0001). Among the patients with FA and barrier dysfunction 10 patients had a structural defect of the intestinal barrier as defined by leakage of fluorescein with multiple cells shedding into the intestinal lumen and exposure of the lamina propria (Watson Grade III) while 16 patients exhibited a functional barrier defect with single epithelial cell shedding accompanied by fluorescein leakage over the intestinal barrier (Watson Grade II). In contrast, of the 11 patients with barrier dysfunction in the non-FA, all were functional barrier defects while no patient in the non-FA group had a structural barrier defect (Fig. 2). Accordingly, the corresponding mean Watson score was significantly higher in all patients with FA than in those without (FA: mean 2.3 ± 0.56 [SD]; no FA: mean 1.33 ± 0.48 [SD], *p* < 0.0001). Accompanied by this, microerosions, defined as an exposure of the lamina propria to the lumen with multiple cells being shed per site, were highly significant more frequent in patients with FA compared to those without: 25 out of 27 patients (92.6%) exhibited microerosions in the terminal ileum whereas only 8 out of 33 patients (24.2%) without FA had microerosions during CLE in the terminal ileum (*p* < 0.0001, Fig. 3).

Based on this significant increase of barrier dysfunction and microerosions in the terminal ileum of patient with FA, we sought to explore the clinical potential of CLE imaging for the diagnosis of FA and hypothesized that visualization of the intestinal barrier might be used as a screening test for in vivo identification of patients with FA. To test this, we calculated diagnostic performances of visualizing barrier dysfunction for the diagnosis of FA. As shown in Table 2, assessing barrier function with pCLE had a sensitivity and specificity for the in vivo diagnosis of FA of 96% and 67%, respectively, with a positive and negative prediction of 70% and 96%, respectively.

**Table 2:** Diagnostic performance of assessing barrier dysfunction in the terminal Ileum by pCLE for the diagnosis of Food Allergy (FA).

	FA present	FA absent	
Barrier dysfunction	26	11	PPV = 70.3% (59.2–79.4%)
No barrier dysfunction	1	22	NPV = 95.7% (76–99.4%)
	Sens = 96.3% (81–99.9%)	Spec = 66.6% (48.2–82%)	

Sens, sensitivity; Spec, specificity.

PPV, positive predictive value; NPV, negative predictive value.

(95% CI).

**Figure 4:**
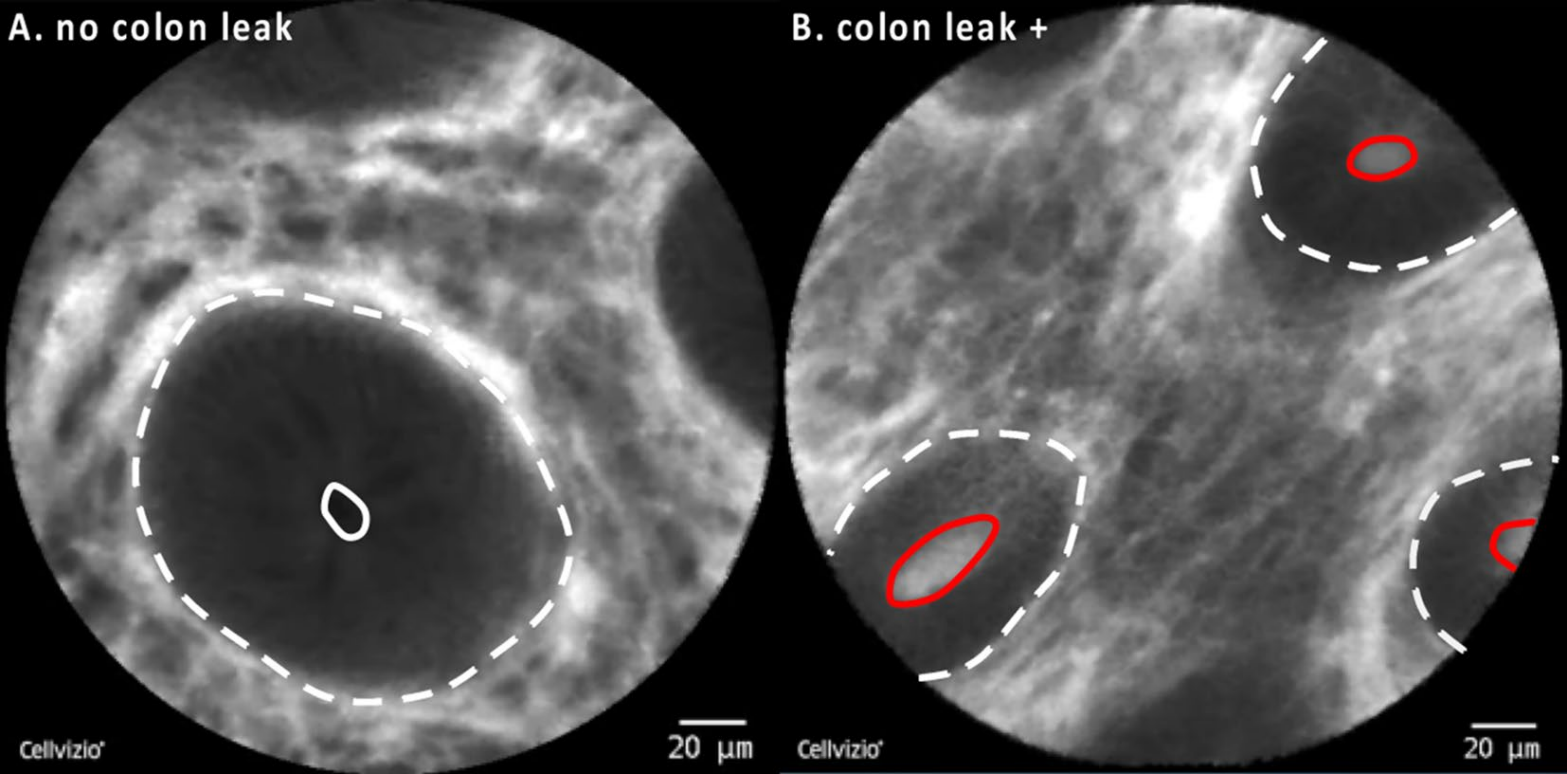
Barrier dysfunction in the colon under CLE imaging. (**A**) Intact epithelial barrier in the colon is characterized by a crypt opening that appears as a dark center in the center of the crypt (white circle). (**B**) During colonic barrier dysfunction, fluorescein leaks into the crypt lumen; therefore, the lumen is brighter than the surrounding epithelium (red circle). Dashed white line: colonic crypts.

Given the high sensitivity of assessing barrier dysfunction in the terminal ileum for the in vivo diagnosis of FA, we then hypothesized that colonic barrier dysfunction might exhibit equal diagnostic performances for the in vivo diagnosis of FA. Using a dichotomous grading of colonic barrier dysfunction as previously described^13,17,18^ (Fig. 4), we found barrier dysfunction during functional imaging with pCLE in 10 out of 27 patients (37%) with FA. In the non-FA group, 6 out of 33 patients (18%) exhibited colonic barrier dysfunction and results between the two groups were not statistically significant (*p* = 0.144). When calculating the diagnostic performance of visualizing barrier dysfunction in the colon with pCLE, a sensitivity of 37% and a specificity of 82% for the diagnosis of FA were obtained with a positive and negative prediction of 62.5% and 61%, respectively (Table 3).

**Table 3:** Diagnostic performance of assessing barrier dysfunction in the colon by pCLE for the diagnosis of Food Allergy (FA).

	FA present	FA absent	
Barrier dysfunction	10	6	PPV = 62.5% (41–80%)
No barrier dysfunction	17	27	NPV = 61.4% (53.3–68.9%)
	Sens = 37% (19.4–57.6%)	Spec = 81.8 (64.5–93.2%)	

Sens, sensitivity; Spec, specificity.

PPV, positive predictive value; NPV, negative predictive value.

(95% CI).

### Cryptometry of the colon with pCLE in patients with and without Food Allergy

**Figure 5:**
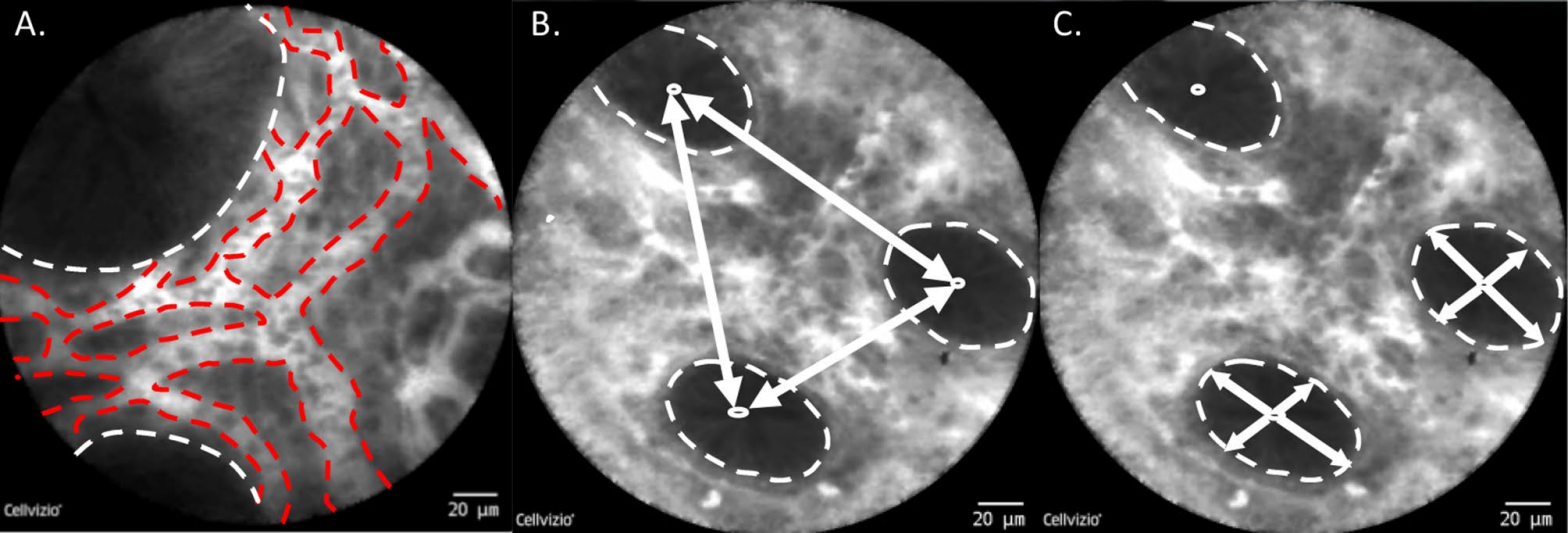
Quantitative Cryptometry of the colonic architecture by CLE. (**A**) Mean, minimal and maximal vessel diameter were measured using the vessel detection plug in of the IC-Viewer which enables automatic detection of the vessels directly from each endomicroscopic recording frame based on fluorescence intensity detection. Detection threshold was manually set at 10 μm for vessel detection. Dashed white line: colonic crypts, red dashed line: vasculature in the lamina propria. (**B**) Intercrypt distance (white arrows) was defined, as the distance between the geometrical centers of neighboring crypts, as defined by the crypt lumen. Distances between all crypts in one images were quantified. (**C**) Total crypt diameter was defined as the distance between epithelial cells constituting the crypts measured in 2 axes crossing the centers of the crypts (white arrows). Parameters in B and C were measured using the IC-Viewer software version 3.8.6 (Mauna Kea Technologies, Paris, France). Dashed white line: colonic crypts.

We further explored whether parameters intrinsic to the colonic architecture (intercrypt distance, total crypt diameter, crypt lumina diameter) and colonic vascularization are different between patients with and without FA (Fig. 5).

As shown in Table 4, intercrypt distance (ICD) was virtually identical in patients with and without FA (mean ICD FA: 93.5 µm; mean ICD no FA: 93.5 µm). Minimum and maximum crypt diameter were 90.4 µm and 127.5 µm in patients with FA and 92 µm and 125 µm in patients without FA, respectively. Crypt lumina diameter was also not significantly changed between with and without FA (with FA: 12.4 µm; without FA: 12 µm).

**Table 4:** Cryptometry and Vasculature in the colon in patient with and without Food Allergy.

	No food allergy (n = 33)	Food allergy (n = 27)	*p* value
**Crypt diameter**			
Mean ± SD	108.4 ± 26.9	109.0 ± 30.0	0.73
Minimum, mean	91.8	90.4	
Maximum, mean	125.1	127.5	
**Intercrypt distance (ICD)**			
Mean ± SD	93.5 ± 34.5	93.5 ± 34.4	0.99
Minimum, mean	69.2	68.2	
Maximum, mean	117.8	118.8	
**Crypt lumen diameter**			
Mean ± SD	12.0 ± 7.8	12.4 ± 5.5	0.59
**Vessel diameter**			
Mean ± SD	12.5 ± 1.1	12.4 ± 1.2	0.28
Minimum, mean	5.4	5.4	
Maximum, mean	18.9	18.9	

With a mean of 12.4 µm and 12.5 µm, mean vessel diameter was also not significantly different between patients with and without FA. Similarly, minimal and maximal vessel diameter was virtually identical between patients with FA and those without.

## Discussion

Since its introduction to the market more than a decade ago, CLE has emerged as a technology that allows not only for microscopy imaging in realtime in the GI tract but is the only imaging modality to date to functionally assess the integrity of the intestinal barrier. Here, we explored the clinical potential of this advanced in vivo imaging technology in patients with suspected food allergy (FA). CLE was highly sensitive for the in vivo diagnosis of FA and detected intestinal barrier dysfunction significantly more frequent in patients with FA compared to controls. Further, with a high negative prediction of > 90%, our results indicate that CLE can be used a screening test that can help in stratifying patients into those in which further diagnostic work-up for suspected FA should be performed and those that have a low likelihood of suffering from food allergy.

Assessing barrier dysfunction has been first performed in patients with inflammatory bowel disease (IBD) and from early landmark studies in the field, a semiquantitave grading of barrier dysfunction has been developed^14^. This so-called Watson Grading has subsequently been utilized by several other trials^12,13,15,16^, thereby suggesting that this categorical distinction into intact barrier function as opposed to a functional or structural barrier defect allows for reproducible and reliable assessment of the integrity of the intestinal barrier. Importantly, as shown across studies, barrier dysfunction in IBD can predict the occurrence of relapse or complicated disease behaviour in IBD patients in remission^13,14,16,17^. Recently, a prospective study was able to directly link increased intestinal permeability to persistence of symptoms in IBD patients. As shown in 110 consecutive IBD patients with mucosal healing on white light endoscopy, impaired barrier function in the terminal ileum was strongly associated with ongoing bowel symptoms and increased permeability correlated with increased severity of diarrhea^12^.

Apart from that, early pilot studies have already described an increased density of epithelial gaps in the terminal ileum of patients with irritable bowel syndrome (as measured by pCLE) compared with healthy controls^20^. However, although the authors did not assess the functional relevance at that time, it was already speculated that increased epithelial cell extrusion, as measured by CLE, may represent a potential mechanism for altered intestinal permeability in IBS patients. More recent studies provide first insights into the functional relevance of this. As elegantly shown in a landmark pilot study, exposure to candidate food antigens in IBS patients with a suspected food intolerance caused immediate breaks and increased intervillous spaces in the intestinal epithelial barrier and these changes were directly visualizable in real time by CLE when the antigen was administered to the duodenal mucosa^21^. Further, when patients were subsequently put on exclusion diet of the food antigen they reacted to during live CLE imaging, patients experienced significant relief or even cessation of symptoms^21^. This study was the first to show that intestinal barrier dysfunction occurs as a direct consequence of mucosal exposure to certain allergen and can be dynamically monitored by CLE.

In a recent prospective study 108 patients with IBS received challenges with each of 4 common food components via the endoscope, followed by CLE in the duodenum^19^. Importantly, classical food allergies were excluded prior to allergen exposure during CLE by negative results from immunoglobulin E serology analysis and skin tests for common food antigens. Using this approach it was shown that more than 50% of patients reacted with immediate disruption of the intestinal barrier and fluorescein leakage upon exposure to food antigens, the majority of which reacted to wheat^19^. These findings were paralleled by increased expression of claudin-2 and decreases in occludin in duodenal biopsies and a trend towards increased eosinophil degranulation suggesting that at least 50%–60% of IBS patients may have a nonclassical food allergy in which CLE can be used to dynamically monitor impairment of the intestinal barrier^19^.

While the latter two studies provide proof-of-principle that CLE can visualize occurrence of dysfunction upon allergen exposure, the broad applicability of this approach with sequential exposure of the mucosa with 5 different antigens and CLE examinations after each exposure might be limited, especially against the background that food allergy or self-reported adverse reactions to food are prevalent diseases, thereby limiting the possibility to screen a large percentage of the population with this approach.

Our approach was based on the concept that a screening test for food allergy should ideally be applicable without the necessity for prior preparation (e.g. exclusion diet) and easy and fast to perform, thereby allowing to directly examine large numbers of patients. Based on these considerations, we performed a cross-sectional study in which consecutive patients presenting with symptoms compatible with FA were included. Importantly, since diagnosis of food allergy was not established at the time of endoscopy and CLE, all patients were under regular, and therewith potentially allergen-containing, diet. As shown by our results, presence of barrier dysfunction in the terminal ileum was significantly more frequent in patients in which diagnosis of food allergy was established during the subsequent work-up as compared to those patients in which food allergy was ruled out (96% vs. 33%, respectively). Remarkably, calculating the diagnostic performance of visualizing barrier dysfunction with pCLE for the diagnosis of food allergy revealed a sensitivity of 96% and negative prediction of 96%. Especially the high negative prediction in our pilot study is a finding that, if confirmed in larger trials, holds the potential to turn into clinical decision making. In that regard it can be envisioned to use cross-sectional CLE imaging of the intestinal barrier as a screening test that identifies patients in which further work-up for food allergy is warranted (e.g. those with positive CLE findings) whereas those without signs of barrier dysfunction have a low likelihood of suffering from food allergy. Concretely, this would allow for a step-wise approach in which patients with symptoms compatible with food allergen would first receive an initial cross-sectional pCLE imaging of the small bowel that can be readily incorporated into routinely performed procedures (EGD, colonoscopy). In case of CLE positivity (as characterized by the presence of barrier dysfunction) during this initial evaluation, these patients will be subject to further more time-consuming and sophisticated CLE-examinations with subsequent exposure of the mucosa to different allergens to identify the respective allergen and/or to diagnose atypical food allergen, as shown by Fritscher-Ravens and colleagues^19,21^.

Limitations of our study also need to be addressed one of which is the single center setting. Although it seems clear that larger studies in a multicentric setting are highly warranted, CLE is a technology that is not widely distributed and mainly available at selected academic centers, so that also other studies in the field were performed at single centers with comparable or even lower numbers of patients included^19–21^. Further, our patients were included under regular diet (therewith under potential antigen containing diet) and it would have been desirable to analyze whether the observed barrier dysfunction in this cross-sectional study is reversible during follow-up examinations under exclusion diet.

In summary, our results show for the first time that intestinal barrier dysfunction is significantly more frequent in patients with FA under regular diet compared to those without. Further, we demonstrate that assessing the intestinal barrier with CLE is highly sensitive for the in vivo diagnosis of FA and has a high negative predictive value to rule out FA. Therefore, CLE-based imaging of the intestinal barrier during routine colonoscopy might help for stratifying patients with suspected FA for further diagnostic work-up.
